# Statistical test for $\Delta \rho_{\mathrm{DCCA}}$: Methods and data

**DOI:** 10.1016/j.dib.2018.03.080

**Published:** 2018-03-22

**Authors:** E.F. Guedes, A.A. Brito, F.M. Oliveira Filho, B.F. Fernandez, A.P.N. de Castro, A.M. da Silva Filho, G.F. Zebende

**Affiliations:** a Computational Modeling Program, SENAI CIMATEC, Salvador, Bahia, Brazil; b Federal Institute of Paulo Afonso, Bahia, Brazil; c Estácio de Sá University, Gilberto Gil Campus, Salvador, Bahia, Brazil; d Municipal Foundation Egberto Costa, Feira de Santana, Bahia, Brazil; e Jorge Amado University Center, Bahia, Brazil; f Earth Science and Environment Modeling Program, State University of Feira de Santana, Bahia, Brazil; g Department of Physics, State University of Feira de Santana, Bahia, Brazil

## Abstract

In this paper the algorithm for $\Delta \rho_{\mathrm{DCCA}}$ statistical test (Guedes et al., 2018) [1] is presented. Our test begins with the simulation of four time series pairs, by an ARFIMA process. These time series has N=250, 500, 1000, and 2000 points, see Guedes et al. (2018) [1]. The probability distribution function (PDF) is made available for all 10,000 samples, that start from the original time series, in supplementary material.

**Specifications Table**

**Table:** 

Subject area	Physics and Astronomy
More specific subject area	General physics and methods
Method name	$\Delta \rho_{\mathrm{DCCA}}$ Statistical test
Reference of the original paper	〈https://doi.org/10.1016/j.physa.2018.02.148〉
Type of data	Zip File (Deltarhodata.zip)
Data format	ASCII (Raw and analyzed)
Data Accessibility	Data are accessible within the article

**Value of the data**

• A robust test to analysis cross-correlation is important.
• However, for $\Delta \rho_{\mathrm{DCCA}}$, there is not a statistical test.
• Here, we presented the algorithm to test the significance of $\Delta \rho_{\mathrm{DCCA}}$.
• Finally, the probability distribution function (PDF) for this test is found, as a supplementary material (Deltarhodata.zip), and this PDF allows that other researchers extend yours analyses.

## 1 Data

**Fig. 1:**
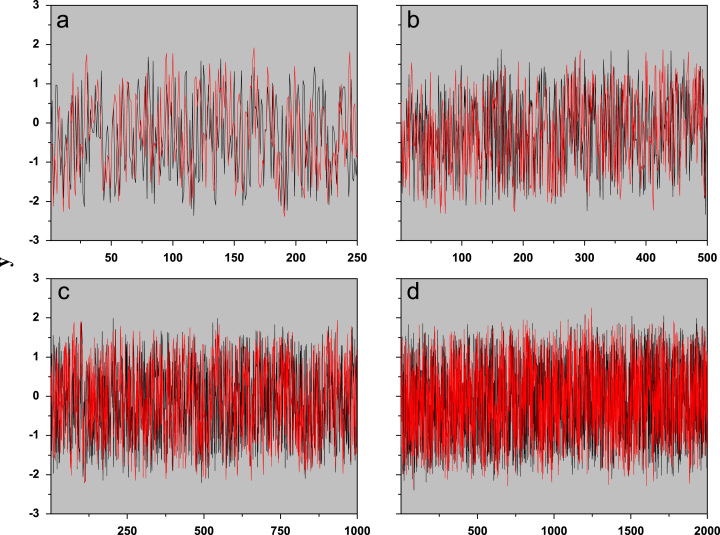
Original time series (pairs) produced by an ARFIMA process, with: (a) N=250, (b) N=500, (c) N=1000, and (d) N=2000.

The test starts with four time series pairs with N=250, N=500, N=1000, and N=2000 points, produced by an autoregressive integrated moving average process (ARFIMA) [2], [3], see Fig. 1. These time series initially are useful in modeling time series with long memory, and are found in the supplementary material as ASCII file: {(af2501.txt; af2502.txt), (af5001.txt;af5002.txt), (af10001.txt;af10002.txt), (af20001.txt;af20002.txt)}. After this time series simulation the algorithm for statistical test is presented below in the Section 4.

## 2 Experimental design, materials, and methods

**Fig. 2:**
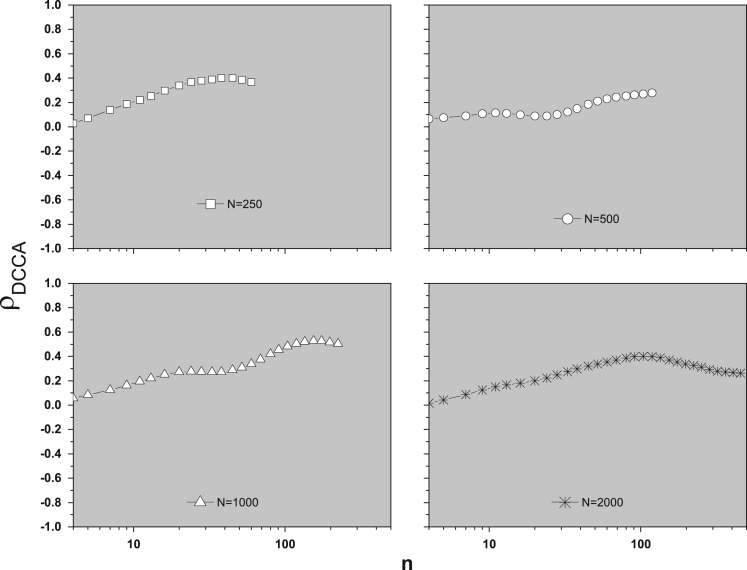
$\rho_{\mathrm{DCCA}}$ as a function of *n*.

Initially, in the sense to verify the cross-correlation between these original time series (raw data), we applied the Detrended cross-correlation coefficient, $\rho_{\mathrm{DCCA}}$ [4], see Fig. 2.

**Fig. 3:**
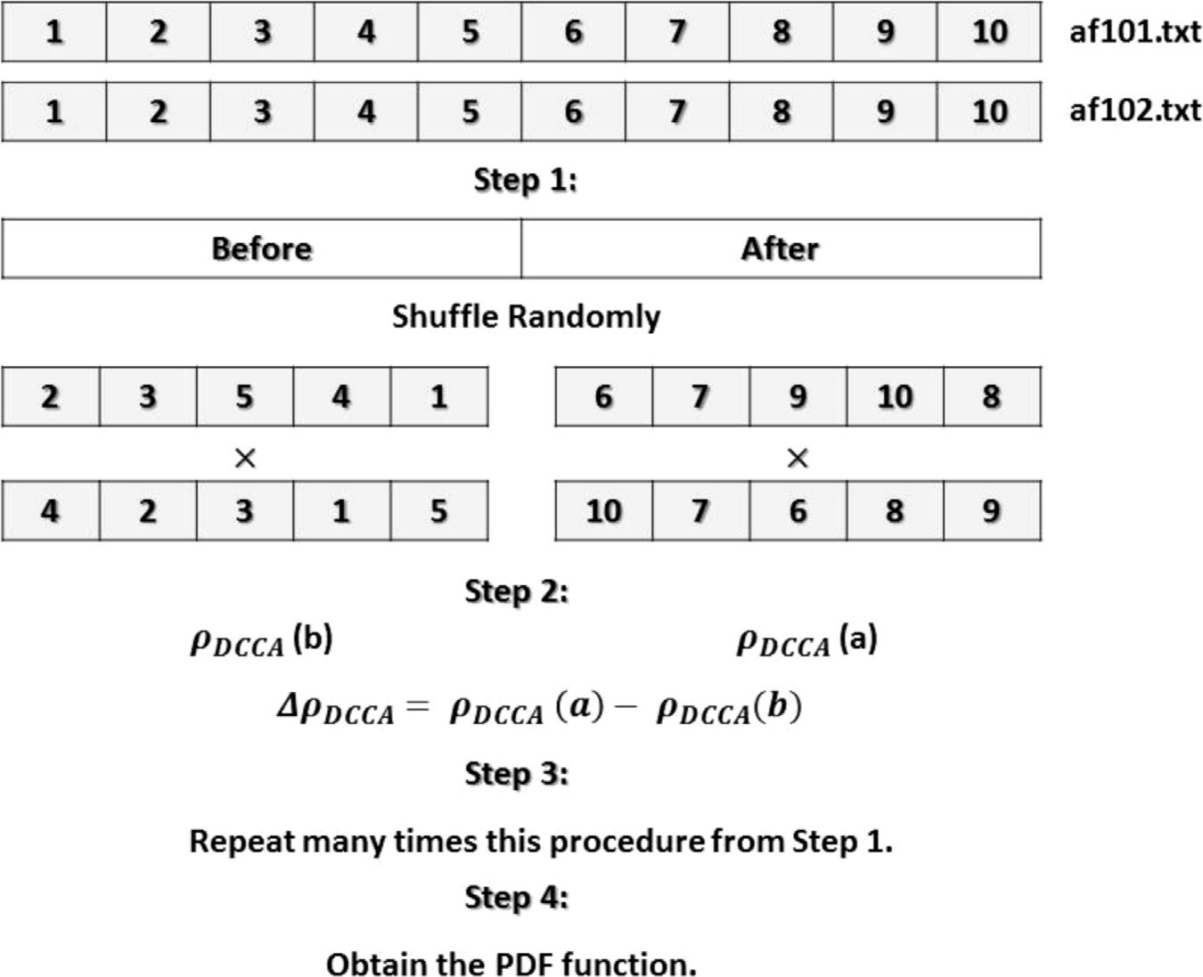
The algorithm procedure.
(b) Estimate $\rho_{\mathrm{DCCA}}(n)$ (each part) and their difference $\Delta \rho_{\mathrm{DCCA}}(n)$ [1];
(c) Repeat this procedure many (10,000) times from Step (a);
(d) And, finally obtain the PDF function of $\Delta \rho_{\mathrm{DCCA}}(n)$.

Thereafter, from the original signal we:

(a) Split these time series into two (before/after) and we shuffle randomly these pairs (see Fig. 3);

See the supplementary material (Deltarhodata.zip).

## Appendix B Supporting Information

Application 2
