# Supplementary material for: Statistical test for ΔρDCCA: Methods and data
Source: Data Brief. 2018 Mar 22;18:795–8. doi: 10.1016/j.dib.2018.03.080 (PMC6103976; doi:10.1016/j.dib.2018.03.080)
Supplement: Application 1 [file mmc1.pdf]

Conflicts of Interest

The authors certify that they have NO affiliations with or involvement in any organization or entity with any financial interest (such as honoraria; educational grants; participation in speakers' bureaus; membership, employment, consultancies, stock ownership, or other equity interest; and expert testimony or patent-licensing arrangements), or non-financial interest (such as personal or professional relationships, affiliations, knowledge or beliefs) in the subject matter or materials discussed in this manuscript.

The authors
